# Editorial: Tertiary Lymphoid Organs (TLOs): Powerhouses of Disease Immunity

**DOI:** 10.3389/fimmu.2017.00228

**Published:** 2017-03-06

**Authors:** Changjun Yin, Sarajo Mohanta, Pasquale Maffia, Andreas J. R. Habenicht

**Affiliations:** ^1^Institute for Cardiovascular Prevention, Ludwig Maximilians University of Munich, Munich, Germany; ^2^German Centre for Cardiovascular Research (DZHK), Munich Heart Alliance, Munich, Germany; ^3^Centre for Immunobiology, Institute of Infection, Immunity and Inflammation, University of Glasgow, Glasgow, UK; ^4^BHF Centre for Excellence in Vascular Science and Medicine, College of Medical, Veterinary and Life Sciences, University of Glasgow, Glasgow, UK; ^5^Department of Pharmacy, University of Naples Federico II, Naples, Italy

**Keywords:** tertiary lymphoid organs, disease immunity, antigen, autoimmunity, autoinflammation, immune tolerance, non-resolving peripheral tissue inflammation, dichotomies of immune responses

**Editorial on the Research Topic**

**Tertiary Lymphoid Organs (TLOs): Powerhouses of Disease Immunity**

Tertiary lymphoid organs (TLOs) arise in peripheral tissues (1) of adult organisms in response to non-resolving inflammation (2) including chronic infection, allograft rejection, cancer, autoimmune diseases, and a large number of other pathologies. One denominator of many—but certainly not all—of these conditions may be the presence of (auto)antigens that are recognized by the immune system as non-self. This notion—though unproven—is consistent with what we would like to call the *antigen-driven TLO hypothesis*. However, for many TLO-associated diseases, the presence of antigen-triggered immune responses—let alone antigen-triggered disease-causing immunity—has not been demonstrated (3). We therefore consider an alternative sequence of events, i.e., that TLOs arise in response to an inflammatory environment in the absence of antigen(s). The latter school of thought may be recapitulated as *inflammation-driven TLO hypothesis*. It is conceivable that chronic inflammation may be initiated by any form of unspecific, antigen-independent tissue injury, leading, in the second phase, to protracted inflammatory tissue reaction that may ultimately unmask previously cryptic epitopes. However, it is not known at which stage of chronic inflammation TLOs are triggered. During the third stage, when breakdown of peripheral tolerance reaches a critical level, autoreactive T- and/or B-cells may be generated to cause clinically overt organ destruction. Of course, any of these scenarios generate proinflammatory and immunosuppressive T- and B-cells yielding complex dichotomically acting lymphocyte subsets that may initially coexist for long periods of time without tissue damage. Cytokines associated with inflammation may be sufficient in many instances to initiate and later to shape TLO phenotypes. Again, critical checkpoints in the immune system leading to T/B lymphocyte clusters remain to be identified, although the formation of lymphorganogenic chemokines has been identified as drivers of both secondary lymphoid organs during embryogenesis and TLO neogenesis in adult organisms. The presence of antigen, however, is apparent in infectious diseases, allogeneic transplant rejection, some forms of cancers, and *bona fide* autoimmune diseases such as *Myasthenia Gravis* and *Basedow’s Disease*. Thus, the search for and identification of antigen(s) as the potential driving forces of TLO-dependent autoimmunity continue. New approaches and technologies including single-cell transcriptome analyses (4), next-generation sequencing of the T- and B-cell receptor repertoires (5), improved large-scale autoantigen detection technologies (6), tissue clearing methods (7), and translational research from experimental systems into human diseases will all be important to make progress.

Tertiary lymphoid organs are striking illustrations of the amazing degree of plasticity of the immune system in response to non-resolving peripheral tissue inflammation. Recent research has uncovered key common features between secondary lymphoid organogenesis and TLO formation. However, the various types of TLOs also reveal some disease-specific features, which may ultimately determine whether the associated immune responses are harmful or protective. Such disease-specific characteristics may arise through one of several mechanisms including organ specificity and the nature of tissue damage. A major challenge for future research is to identify both the common and the specific features of individual TLO-associated diseases, with a view to develop new selective immune-based therapies. In an ideal case scenario, such therapeutics should interfere with TLO immunity (promotion or suppression) without compromising the systemic immune response and the integrity of the surrounding tissue. While identification of shared mechanisms has made major advances in recent years, disease-specific alterations of TLO immunity are less well understood. Studies on the peripheral plasticity of TLO characteristics should focus on CD4^+^ T cells (8); microglia cells in the central nervous system (9); the biology of epithelia and tissue environment in cancer (10); immunoglobulin-like receptors (11); innate immune cells (12); immune tolerance mechanisms (13); B-cell subtype plasticity in autoimmunity (14); identification of transcriptional and epigenetic circuits in dendritic cells, other immune cells, and the mesenchyme (15); identification of special phenotypes of tissue-specific monocytes/macrophages (16); meningeal Th-17 cell (17); intestinal microbiota (18); the various forms of lymphoid tissue organizer cells (19), and lymphoid tissue inducer cells (20). Advances in these areas are likely to yield new insight into the pathogenesis of chronic inflammation and may pave the way for the design of novel tissue- and/or disease-specific therapeutic approaches.

The recent surge of interest in the TLO microcosm of disease immunity is remarkable as very little is known about the impact of these lymphocyte aggregates on disease progression: How and when TLOs are triggered? What distinct tasks do they have—located so close to diseased tissues when compared to the more distant secondary lymphoid organs? Are they critically important for disease progression and—if they are—what are the underlying mechanisms? How do they connect to the tissue-draining lymph nodes and lymphatics? Are they beneficial, injurious, or both, depending on the context and stage of disease, anatomical location, and other factors? Can they be exploited or even artificially constructed for therapeutic purposes? Are there ways to disrupt them or enhance their activity without interfering with the systemic immune response? These are only a few of the questions that are being addressed in various disciplines. Until these fundamental issues will have been resolved, the interest in TLOs will most likely intensify. Hence, this research topic assembles scientists who agree on the premise that it is time to study these enigmatic lymphocyte aggregates over a broad range of angles.

This research topic is organized into two major sections as follows.

*Section 1 covers the structures and cellular composition of TLOs and their development and function*. It is introduced by Nancy Ruddle (Yale University) who provides an overview on functional and regulatory features of high endothelial venules (HEVs) and the lymphatic system in TLOs; Nancy emphasizes the importance of HEVs in the recruitment of naïve and central memory lymphocytes and of lymph vessels to transport antigen and serve as an entry site for antigen-presenting cells and lymphocytes. Ann Ager (Cardiff University) also discusses blood vessels and HEVs as critical regulators of lymphoid organ development and function. In particular, this review focuses on the role of vascular addressins in the regulation of lymphocyte trafficking and stresses the role of CD11c^+^ dendritic cells to regulate addressin expression in HEVs. Francesca Barone et al. (University of Birmingham and University of Lausanne) discuss the complex issue of stromal fibroblasts in shaping TLOs; Francesca proposes that these cells may represent a novel therapeutic target in chronic inflammation. Jan Kranich (Ludwig-Maximilians-University Munich) and Nike Julia Krautler (ETH Zurich) cover the area of follicular dendritic cells in shaping the B-cell antigenome. Jan and Nike highlight the origin and role of follicular dendritic cells in the capture and retention of antigen, thereby allowing B cells to undergo affinity maturation in germinal centers. Catherine Hughes et al. (University of Glasgow) assess the area of TLO antigen presentation and antigen-presenting cells. Gareth Wyn Jones et al. (Cardiff University) evaluate and discuss the complex interaction of immune cells as drivers in TLO development. James A. Butler et al. (University of York) provide an overview on their unconventional approach of model-driven experimentation to understand TLO neogenesis and function. Finally, Katrijn Neyt et al. (Ghent University) complement this section by providing original data on the role of interleukin 1 in TLO neogenesis.

*Section 2 covers prototypical examples of an expanding number of clinically significant diseases in which TLOs have been observed*. Alice Koenig and Olivier Thaunat (University of Lyon) discuss TLOs in chronic transplant rejection; Alice and Olivier challenge the traditional view that TLOs may contribute to transplant rejection by pointing out the recent finding that TLOs have been described in stably accepted organ grafts. Elisa Corsiero et al. (Queen Mary University of London) review ectopic lymphoid structures as powerhouses of autoimmunity, and Meike Mitsdoerffer and Anneli Peters (Technical University of Munich and Max Planck Institute of Neurobiology Munich) focus attention on central nervous system autoimmunity. Changjun Yin et al. (Ludwig-Maximilians-University Munich) discuss the structures and possible impacts of artery TLOs in atherosclerosis. Manuela Buettner and Matthias Lochner (University of Hannover) summarize the special features, development, and function of TLOs in the small intestine and colon; Nobuyoshi Hiraoka et al. (National Cancer Center Institute Tokyo) cover the expanding area of TLOs in cancer tissues. Eoin Neil McNamee and Jesus Rivera-Nieves (University of Colorado and University of California San Diego) discuss the roles of TLOs in inflammatory bowel disease as potentially protective or disease-promoting immune cell aggregates. Aliyah M. Weinstein and Walter J. Storkus (University of Pittsburg) summarize what is currently known on the biosynthesis and functional significance of peripheral node addressin in cancer-associated TLOs. Ji Young Hwang et al. (University of Alabama at Birmingham) point out the unique features of inducible bronchus-associated lymphoid tissue as protective immune aggregates in the lung. Jorge Caamaño and Sara Cruz-Migoni (University of Birmingham) summarize the recent discovery of fat-associated lymphoid clusters and their potential role in bridging metabolism and inflammation in adipose tissue. Marc Clement et al. (University of Paris Diderot) provide original observations on TLOs in Takayasu Arteritis. The section on TLOs in diseases is complemented by Catherine Sautes-Fridman et al. (Cordeliers Research Center Paris) on the epidemiology of tertiary lymphoid structures in cancers. The research topic ends with an original contribution by Yuka Kobayashi and Takeshi Watanabe (Tazuke-Kofukai Medical Research Institute Osaka) on their work on the *in vivo* generation of artificially constructed TLOs and their function in attempts to exploit TLOs for future therapeutic purposes.

We hope that this research topic and E-book will help to *start spreading the news* on TLOs to accelerate information on and interest in the important issue of the functional impacts of these still largely enigmatic disease-restricted lymphocyte aggregates.

## Author Contributions

All authors listed have made substantial, direct, and intellectual contribution to the work and approved it for publication.

## Conflict of Interest Statement

The authors declare that the research was conducted in the absence of any commercial or financial relationships that could be construed as a potential conflict of interest.
